# *Juglans regia* and *Pfaffia paniculata* extracts: implications for periodontal disease treatment and correlation with Alzheimer’s risk

**DOI:** 10.3389/fcimb.2025.1585438

**Published:** 2025-05-16

**Authors:** Diego Garcia Miranda, Florence Carrouel, Nina Attik, Gabriela Ferraz Araujo, Nicole Fernanda Dos Santos Lopes, Maria Cristina Marcucci, Flavia Pires Rodrigues, Giovanna Arruda Caires, Hugo Vigerelli, Bruno Henrique Godoi, Cristina Pacheco-Soares, Lucas de Paula Ramos

**Affiliations:** 1 Laboratory Health Systemic Process - P2S, UR4129, Faculty of Medicine Laennec, University Claude Bernard Lyon 1, University of Lyon, Lyon, France; 2 Department of Biosciences and Oral Diagnosis, Institute of Science and Technology, São Paulo State University, São José dos Campos, Brazil; 3 Multimaterials and Interfaces Laboratory, CNRS UMR 5615, University Claude Bernard Lyon 1, University of Lyon, Lyon, France; 4 Faculty of Medicine and Health, School of Dentistry, Oral Biology Division, University of Leeds, Leeds, United Kingdom; 5 Laboratory of Genetics, Butantan Institute, São Paulo, Brazil; 6 Laboratório de Bioquímica, Instituto Butantan, São Paulo, Brazil; 7 Laboratory of Cell Compartment Dynamics, Institute of Research and Development, University of Vale do Paraíba, São José dos Campos, Brazil; 8 School of Dentistry, Federal University of Alfenas—UNIFAL, Alfenas, Brazil

**Keywords:** *Porphyromonas endodontalis*, *Porphyromonas gingivalis*, neurodegenerative disease, dementia, herbal medicine, gram-negative anaerobes, inflammation, antimicrobial agents

## Abstract

Periodontal disease (PD) is a significant global health concern, affecting approximately 19% of the world’s population. It is one of the most prevalent diseases today, causing substantial socio-economic impacts and diminished quality of life. Recent research has also revealed a potential link between PD and Alzheimer’s disease. This study investigated the antimicrobial effects of *Juglans regia* and *Pfaffia paniculata* extracts against *P. endodontalis* and *P. gingivalis*, bacteria that cause PD and are related to Alzheimer’s risk. The study also assessed the impact of these extracts on macrophage metabolic activity, pro- and anti-inflammatory cytokine expression, and genotoxicity. The phytochemical analysis of the extract was carried out first. Antimicrobial activity was performed using the M11-A7 protocol (CLSI) for planktonic cultures on monotypic biofilms matured for 168 hours in anaerobiosis. Cell viability analysis was carried out using MTT on mouse macrophages (RAW 264-7), as well as genotoxicity assessment using micronuclei. The anti-inflammatory activity was evaluated using ELISA method, checking the cytokines IL-6, IL-1B, TNF-alpha, IL-17 and IL-10. Phytochemical analysis revealed the presence of Miquelianin, Regiolone and Gallic Acid in *J. regia* extract. For the *P. paniculata* extract, we identified the glycosides Pfaffoside C, Pfaffoside A, 3-O-β-D-glycopyranosyl-oleanolic acid and Beta-ecdysone. Antimicrobial activity revealed a MBC of 1.73 for the extract of J. regia and 0.48 for *P. paniculata* against *P. endodontalis* and *P. gingivalis*. All biofilms were reduced by more than 89% after treatment with the extracts for 5 min. Cytotoxicity evaluations revealed that cell viability remained above 50% at concentrations up to 0.216 mg/ml for *J. regia* and 0.015 mg/ml for *P. paniculata*. Neither extract exhibited genotoxicity. Furthermore, both demonstrated anti-inflammatory activity by promoting the production of the cytokine IL-10. In conclusion, the antimicrobial and anti-inflammatory activities of *J. regia* and *P. paniculata* extracts suggest their potential as treatments for oral dysbiosis, which may contribute to a reduced risk of neurodegenerative diseases.

## 1 Introduction

In recent years, periodontal disease (PD) has emerged as a significant global health concern. According to the World Health Organization, PD affects approximately 19% of the global population, making it one of the most prevalent diseases in the world today (Word Health Organization, 2022). Beyond its high prevalence, PD imposes significant socio-economic burdens, straining healthcare systems and reducing the quality of life for those affected (Jin et al., 2016).

The development and progression of PD is strongly influenced by specific microorganisms in the oral cavity (Gazil et al., 2022). Among them, *Porphyromonas endodontalis* and *Porphyromonas gingivalis*, have been extensively studied for their detrimental effects on both oral and systemic health (Baima et al., 2023; Villalobos et al., 2022; Li et al., 2023; Rao and Kumar, 2023). These Gram-negative anaerobic bacteria are commonly found in the subgingival plaque of individuals with PD, predominantly linked to endodontic infections, including periapical abscesses and orofacial odontogenic infections. Among Porphyromonas species, *P. gingivalis* is the most extensively studied, recognized as a keystone pathogen in periodontitis and implicated in systemic inflammatory conditions (Dahlén and Leonhardt, 2006; Villalobos et al., 2022; Brinar et al., 2023; Li et al., 2023).

Recent researches have revealed a potential connection between PD and Alzheimer’s disease (AD) (Tang et al., 2023; Li et al., 2024; Qiu et al., 2024; Rubinstein et al., 2024). Alzheimer’s disease is a progressive neurodegenerative disorder that affects millions of individuals worldwide (Li et al., 2022). Characterized by neuroinflammation with features suggestive of an infectious etiology, including microglial activation, inflammasome upregulation, complement system engagement, and dysregulated cytokine expression. Although microbial pathogens have been detected in AD (Borsa et al., 2021; Li et al., 2022; Zhang et al., 2023). Studies demonstrated that individuals with PD may be at increased risk of developing Alzheimer’s disease, suggesting a possible link between these two conditions (Borsa et al., 2021; Kaliamoorthy et al., 2022; Zhang et al., 2023). Emerging evidence highlights the role of oral microorganisms, such as *P. gingivalis*, in Alzheimer’s disease pathogenesis (Tang et al., 2023; Li et al., 2024). PD, characterized by chronic inflammation and microbial dysbiosis, may contribute to systemic inflammation and the release of pro-inflammatory mediators. This inflammatory environment could then impact on the central nervous system, potentially influencing the progression of Alzheimer’s disease (Jungbauer et al., 2000; Jin et al., 2021; Zhang et al., 2023).

Furthermore, the treatment of PD raises a number of challenges. One of the main difficulties in treating PD is the antimicrobial resistance exhibited by *P. gingivalis* and *P. endodontalis* other pathogenic bacteria (Ardila et al., 2023; Ng et al., 2023). This antimicrobial resistance hinders the effectiveness of traditional treatment methods, such as antibiotic therapy, and calls for the exploration of alternative therapeutic approaches.

Phytotherapy, the use of plants and their derivatives for therapeutic purposes, has gained attention as a potential treatment for PD (Gawish et al., 2023). Phytotherapics compounds, derived from plants, possess antimicrobial and anti-inflammatory properties and have shown promise in PD management (Bagheri et al., 2022; Gawish et al., 2023). One such phytotherapic agent is *Juglans regia*, commonly known as walnut. *J. regia* exhibit antioxidant (Mates et al., 2023), anti-inflammatory (Sandu-Bălan Tăbăcariu et al., 2024) and antimicrobial properties (Acquaviva et al., 2021). Farooqui et al (Farooqui et al., 2015). demonstrated the antimicrobial spectrum of its methanolic extract against *Salmonella Typhi*, *Salmonella Paratyphi A, Acinetobacter baumannii, Klebsiella pneumoniae, Pseudomonas aeruginosa, Helicobacter pylori, Shigella* sp*ecies, Campylobacter jejuni* and *Escherichia coli*, showing a efficacy against multidrug-resistant clinical strains. *Pfaffia paniculata*, also popularly calls Brazilian Ginseng, express a great anti-inflammatory potential (Costa et al., 2015).

The aim of this study was to assess the antimicrobial effects of *J. regia* and *P. paniculata* extracts against *P. endodontalis* and *P. gingivalis*. Additionally, the study investigated the impact of these extracts on macrophage metabolic activity, the expression of pro- and anti-inflammatory cytokines, and potential genotoxic effects.

## 2 Materials and methods

### 2.1 Chemical reagents

Glycolic extract of *Juglans regia* (CAS n°: 84012-43-1; lot: PRODO18746, Mapric Greentech company^®^, São Paulo, Brazil); Glycolic extract of *Pfaffia paniculata* (lot: PRODO19544, Mapric Greentech company^®^); Brucella broth and agar (Becton Dickinson^®^, Franklin Lakes, New Jersey, USA); Hemin (CAS n°: 16009-13-5, purity: 96%, Sigma-Aldrich^®^, St. Louis, USA); Vitamin K (CAS n°: 58-27-5, purity: 99,8%, Sigma-Aldrich^®^); Sterile saline solution (0.9% NaCl solution) (LGC Biotechnology^®^, Cotia, Brazil); Eagle’s medium modified by Dulbecco (DMEM) (LGC Biotechnology^®^); Fetal Bovine Serum (FBS) (Invitrogen^®^, New York, USA); 3-(4,5-Dimethyl-2-thiazolyl)-2,5-diphenyl-2H-tetrazolium bromide powder (MTT) (CAS n°: 298-93-1, purity: 97,5%, Sigma-Aldrich^®^); Dimethyl sulfoxide (DMSO) (CAS n°: 67-68-5, purity: 99,9%, Sigma-Aldrich^®^); 0,12% Chlorhexidine digluconate (Colgate-Palmolive^®^, New York, USA); Ethyl Methane sulfonate (EMS) (CAS n°: 62-50-0, Sigma-Aldrich^®^); Methanol (CAS n°: 67-56-1, purity: 99,8% Synth^®^, Diadema, Brazil); Cytochalasin B (CAS n°: 14930-96-2, purity: 98%, Sigma-Aldrich^®^); Phosphate Buffer Saline (PBS) (Sigma-Aldrich^®^); Fluoroshield™ 4′,6-Diamidino-2-phénylindole dihydrochloride, 2-(4-Amidinophényl)-6-indolecarbamidine dihydrochloride (DAPI) (CAS n°: 28718-90-3, Sigma-Aldrich^®^); Lipopolysaccharides from *Escherichia coli* O111:B4 (LPS) (Sigma-Aldrich^®^); ELISA kit Duo set codes: IL-10 DY417-05, IL-6 DY406-05, TNF-alpha DY410-05, IL-1B DY401-05, IL-17 DY421-05 (R&D Systems^®^, Minnesota, USA); Tween 20 (CAS n° 9005-64-5, Sigma-Aldrich^®^); Bovine serum albumin (BSA) (CAS n° 9048-46-8, Sigma-Aldrich^®^); Sulfuric acid (CAS n° 7664-93-9, purity: 97%, Sigma-Aldrich^®^).

### 2.2 Equipment

Analytical balance (Mettler Toledo^®^, Balance XPR106DUH/A, Columbus, Ohio, USA); Drying and sterilization oven (CQA Química Americana LTDA^®^, Paulinia, São Paulo, Brazil); Stirrer (Miulab^®^, Micro plate shaker MIX-1500, Hangzhou, China);Water bath precision (Termo Fisher Scientific^®^ TSGP02, Waltham, Massachusetts, USA); Spectrophotometer (Lonza Biotek ^®^, ELX808LBS, Winooski, Vermont, USA); Anaerobic chamber (Don Whitley Scientific Limited^®^, Whitley DG250 Workstation, Shipley, West Yorkshire, UK); Ultrasonic homogenizer (Biosystems^®^, LUHS-A10-1C, Curitiba, Parana, Brazil); CO_2_ incubator: (Sanyo^®^, MCO-19AIC(UV)^®^, Osaka, Japan); Fluorescence microscope (Leica Microsystems^®^, DFC310FX, Wetzlar, Hessen, Germany); Electrospray-Ion Trap-Time of Flight (ESI-IT-TOF) (Shimadzu Co., Japan) equipped with a binary Ultra-Fast Liquid Chromatography system (UFLC, 20A Prominence, Shimadzu).

### 2.3 Phytochemical analysis of *J. regia* and *P. paniculata* extracts by ESI-IT-TOF

The glycolic extracts of *J. regia* and *P. paniculata* (commercially obtained, Lots: PRODO18746 and PRODO19544, Mapric Greentech, São Paulo, Brazil) were prepared by percolation using a mixture of 20% (w/w) plant leaf material and 80% (w/w) propylene glycol. Physicochemical and Phytochemical Characterization were analyzed using MALDI-TOF mass spectrometry (Electrospray-Ion Trap-Time of Flight, ESI-IT-TOF, Shimadzu Co., Japan) coupled with an Ultra-Fast Liquid Chromatography (UFLC) system (Shimadzu 20A Prominence). Separation was performed on a C18 column (Discovery C18, 5 μm, 50 × 2.1 mm) with a binary solvent system: Solvent A2: Water/acetic acid (999:1, v/v) and Solvent B2: Acetonitrile/water/acetic acid (900:99:1, v/v/v). The elution gradient was set at 0–40% solvent B2 over 35 min with a flow rate of 0.2 mL/min. Eluates were monitored using a Shimadzu SPD-M20A PDA detector before MS analysis. The interface voltage was adjusted to 4.5 KV and the capillary voltage was 1.8 KV, at 200°C. MS spectra were acquired under positive mode and collected in the 350–1400 m/z. MS/MS spectra were collected in the 50–1950 m/z range.

### 2.4 Determination of the minimum inhibitory concentration and minimum bactericidal concentration of *J. regia* and *P. paniculata* extracts by CLSI M11-A7 protocol

*P. endodontalis* (ATCC 35406) and *P. gingivalis* (ATCC W83) were cultured on enriched Brucella agar supplemented with 1% hemin and 1% vitamin K at 37°C for 48h under anaerobic conditions. Bacterial suspensions were prepared in sterile 0.9% NaCl and adjusted to 1 × 10^8^ CFU/mL using the McFarland standard. To performed the Antimicrobial Susceptibility Testing, Serial two-fold dilutions (1:2 ratio) of *J. regia* (6.92, 3.46, and 1.73 mg/mL) and *P. paniculata* (1.93, 0.96, and 0.48 mg/mL) extracts were prepared in 100 µL Brucella broth in a microplate. Each well was then inoculated with 100 µL of standardized bacterial suspension. After 48h incubation at 37°C, the minimum inhibitory concentration (MIC) was recorded as the lowest extract concentration preventing visible turbidity. Minimum Bactericidal Concentration (MBC) Determination occur with 10 µL aliquot from each well was subcultured onto Brucella agar and incubated for 48h at 37°C. The MBC was defined as the lowest concentration yielding no bacterial growth, confirming bactericidal activity.

### 2.5 Antibiofilm action of *J. regia* and *P. paniculata* extracts

Monotypic biofilms were established using a bacterial inoculum of *P. endodontalis* or *P. gingivalis* at a concentration of 1 × 10^8^ CFU/mL and incubated for seven days. After biofilm formation, the supernatant was discarded, and the biofilms were treated with *J. regia* extract at concentrations of 6.92, 3.46 and 1.73 mg/mL and *P. paniculata* extract at 1.93, 0.96 and 0.48 mg/mL for min. Additionally, 24-hour treatments were applied at concentrations of 3.46, 1.73 and 0.86 mg/mL for *J. regia* and concentrations of 0.96, 0.48 and 0.24 mg/mL for *P. paniculata*. Each experimental group was performed in 10 replicates. To remove the affected bacterial cells, wells were washed with sterilized 0.9% NaCl solution. The biofilms were then disaggregated using an ultrasonic homogenizer set to 25% Power. Aliquots were drawn from the microplates, diluted at 10^−2^, 10^−4^, and 10^−6^, and seeded onto Brucella agar (10 μL per sample). The plates were incubated in anaerobic chamber for 48h before CFU counting.

### 2.6 Metabolic activity assessment of *J. regia* and *P. paniculata* extracts on mouse macrophages (RAW 264.7)

The metabolic activity of *J. regia* and *P. paniculata* extracts was evaluated in RAW 264.7 cells cultured in DMEM containing 4500 mg/L glucose, supplemented with 10% FBS, and incubated at 37°C with atmospheric humidity and 5% CO_2_ for 5 min and 24h.It was assessed using the MTT colorimetric assay, which measures the cellular enzymatic activity. Cells were seeded in 96-well microplates at a concentration of 2 × 10^4^ viable cells per well in 200 µL of DMEM supplemented with 10% FBS and incubated at37°C with 5% CO_2_ for 24h to allow cell adherence. Subsequently, cells were exposed to 10 different concentrations of the extracts for either 5 min or 24h. DMEM with 10% FBS served as the negative control, while chlorhexidine digluconate was used as the positive control.

Following exposure, the MTT assay was performed by adding 100 µL of a 0.5 mg/mL MTT solution (prepared in DMEM with 10% FBS) to each well. The plates were then incubated in the dark at 37°C with 5% CO^2^ for 4h.After incubation, the solution was discarded, and 100 µL of DMSO was added per well. The plates were then incubated for 10 minutes with agitation, and absorbance was measured at 570 nm using a spectrophotometer. Results were expressed as the percentage of cell viability relative to the untreated control (set as 100%).

### 2.7 Micronucleus test of *J. regia* and *P. paniculata* extracts on mouse macrophages (RAW 264.7)

Mouse Macrophage RAW 264.7 cells were cultured at a concentration of 3x10^5^ cells/mL in 96-well microplates with 1 mL of DMEM supplemented with 10% FBS for 24h at 37°C in a 5% CO_2_ atmosphere. Cells were then exposed to *J. regia* and *P. paniculata* extracts, diluted in DMEM with 10% FBS, at concentrations of 0.108 and 0.054 mg/mL for *J. regia* and 0.48 and 0.24 mg/mL for *P. paniculata*. The negative control group received only the culture medium, while the positive control group was treated with EMS (Ethyl Methane sulfonate) at a concentration of 5 mM. All treatments were applied for 24h.

After treatments, cells were washed three times with PBS and incubated with cytochalasin B (6 μg/mL) for 24h at 37°C in a 5% CO_2_ atmosphere. After incubation, cells were fixed in 100% methanol for 20 min and stained with DAPI. The stain was removed after 5 min of contact with the cells, followed by three washes with PBS. Micronuclei were analyzed under a fluorescence microscope at 40X magnification with a total of 2,000 cells evaluated per well.

### 2.8 Evaluation of pro- and anti-inflammatory cytokine production in LPS-stimulated RAW 264.7 macrophages using ELISA

Mouse macrophages (RAW 264.7) were cultured at a concentration of 5x10^5^ cells/mL in 24-well plates for 24h. Then, the supernatant was discarded, and mouse macrophages (RAW 264.7) were stimulated with LPS from *Escherichia coli* at a concentration of 1 µg/mL per well and incubated for 24h at 37°C in a 5% CO_2_ atmosphere. After incubation, the supernatant was collected and stored at -20°C for subsequent quantification of pro-inflammatory cytokines (IL-1β, TNF-α, IL-6, IL-17) and the anti-inflammatory cytokine IL-10 using an ELISA test.

For the ELISA test, 96-well microtiter plates were coated overnight at room temperature with100 µL/well with anti-TNF-α, anti-IL-1β, anti-IL6, anti-IL17 or anti-mouse IL-10. Plates were then washed with PBS containing Tween 20 (PBS-T) and blocked with BSA 0.1% for 1 hour, using 300µL/well. After washing the plates with PBS-T, 100 µL of cell culture supernatants and cytokine standards (for a standard curve) were added in the plate. Tests were performed in duplicate. Following a 2-hour incubation period, the plates were washed with PBS-T, and biotin-labeled detection antibodies specific for TNF-α, IL-1β or IL-10 were added in the volume of 100µL/well. After a further 2-h incubation, the reaction was developed using a chromogenic substrate solution containing hydrogen peroxide, added in a volume of 100µL/well. The colorimetric reaction was stopped after 20 min by adding 2N sulfuric acid at volume of 50µL/well. Optical densities (OD) were obtained using a spectrophotometer with a wavelength of 450 nm.

### 2.9 Statistical analysis

The data obtained was analyzed for normality using the D’Agostino, Shapiro-Wilk and Kolmogorov-Smirnov tests. Data with normal distribution was analyzed using the one-way ANOVA method complemented by Tukey test. Data without normal distribution was analyzed using the Kruskal Wallis test supplemented by Dunn’s. A significance levels: p < 0.0332 (*), p < 0.0021 (**), p < 0.0002 (***), p < 0.0001 (****). Statistical analysis was carried out using GraphPad Prism 9.0 software.

## 3 Results

### 3.1 Composition of *J. regia* and *P. paniculata* extracts

**Figure 1:**
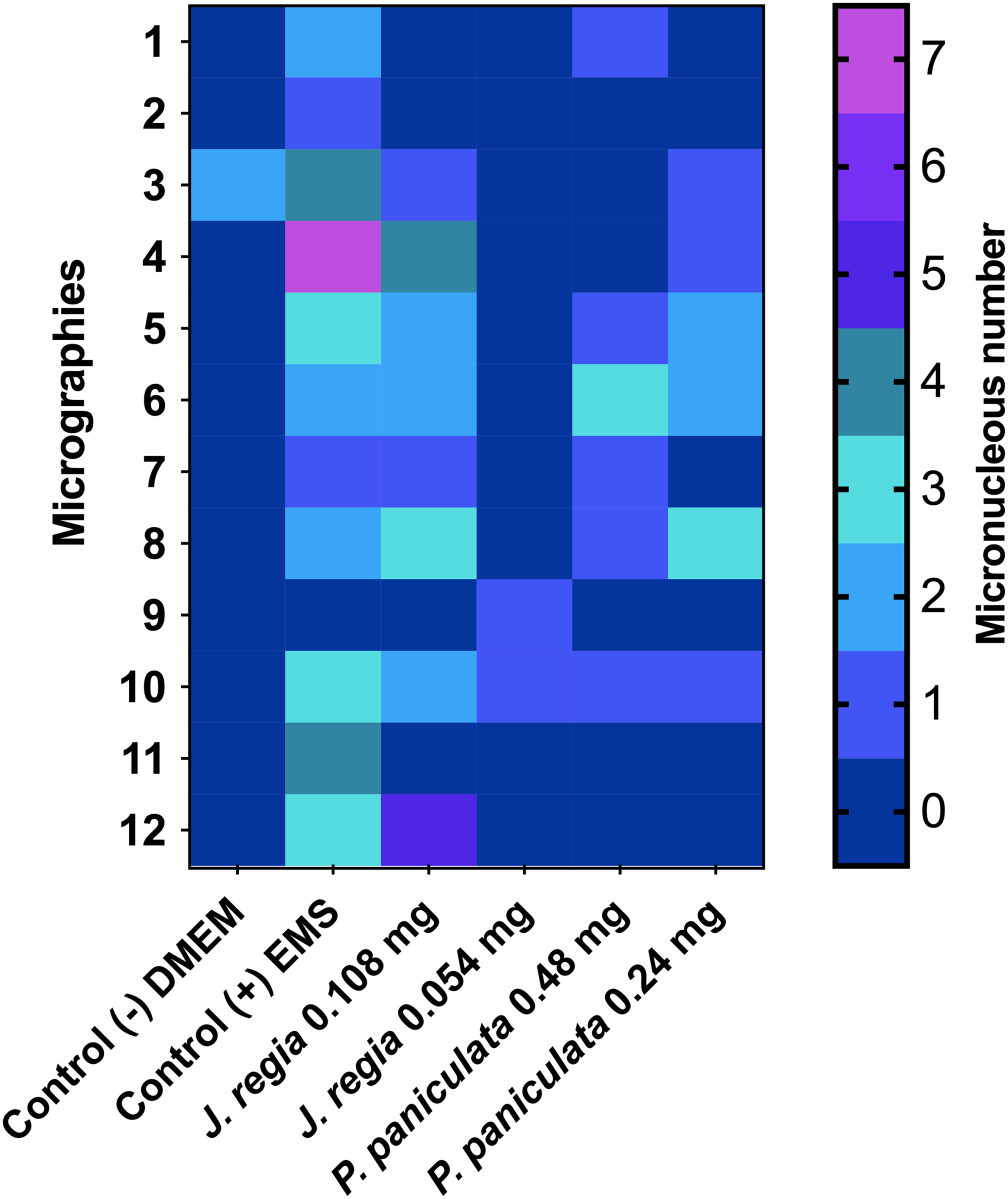
Phytochemical spectrum of the *Juglans regia* glycolic extract.

Legend: The blue spectrum represents the substances present in the *J. regia* extract. The red spectrum indicates the substances present in the matrix used for the technique. The table shows the chemical substances present in the extract.

The phytochemical analysis demonstrated that *J. regia* extract was composed of Miquelianin (peak at 440.1 m/z), Regiolone (peak at 231.25 m/z) and Gallic Acid (peak at 170.12 m/z) (**Figure 1**).

**Figure 2:**
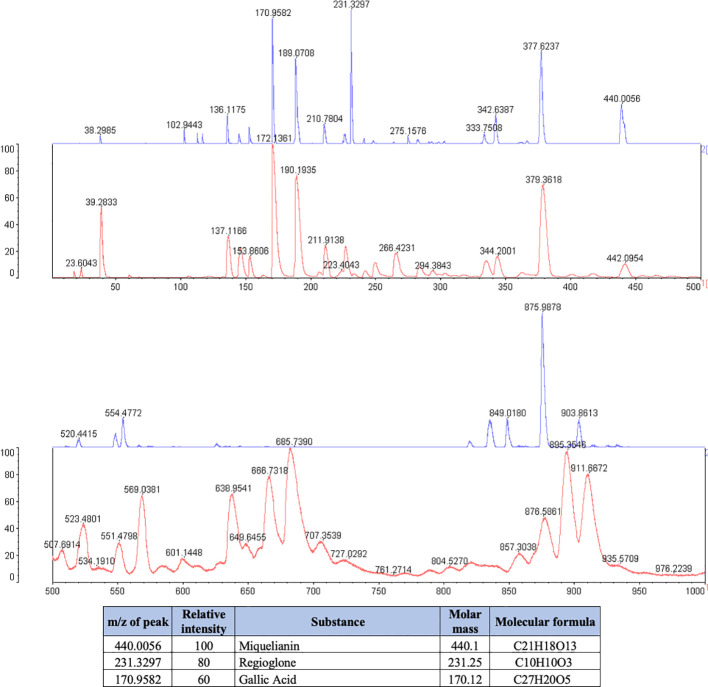
Phytochemical spectrum of the *Pfaffia paniculata* glycolic extract.

Legend: The blue spectrum represents the substances present in the *P. paniclata* extract. The red spectrum indicates the substances present in the matrix used for the technique. The table shows the chemical substances present in the extract.

*P. paniculata* extract was composed of the glycosides Pfaffoside C (peak at 440.39 m/z), Pfaffoside A (peak at 170.16 m/z)3-O-β-D-glycopyranosyl-oleanolic acid (peal at 377.57 m/z) and Beta-ecdysone (peak at 116.07 m/z) (**Figure 2**).

### 3.2 Minimum inhibitory concentration and minimum bactericidal concentration of *J. regia* and *P. paniculata* extracts

The MIC could not be determined due to turbidity of the broth (**Table 1**). The MBC observed was 1.73 mg/mL for *J. regia* and of 0.48 mg/mL for *P. paniculata* against both *P. endodontalis* and *P. gingivalis*.

**Table 1:** MIC and MBC determination for *J. regia* and *P. paniculata* against both *P. endodontalis* and *P. gingivalis*.

Extract	*P. endodontalis*		*P. gingivalis*	
	MIC	MBC	MIC	MBC
*Juglans regia*	nd	1.73 mg/mL	nd	1.73 mg/mL
*Pfaffia paniculata*	nd	0.48 mg/mL	nd	0.48 mg/mL

^nd^Not determined.

### 3.3 Antibiofilm activity of *J. regia* and *P. paniculata* extracts on *P. endodontalis* and *P. gingivalis* monotypic biofilms

**Figure 3:**
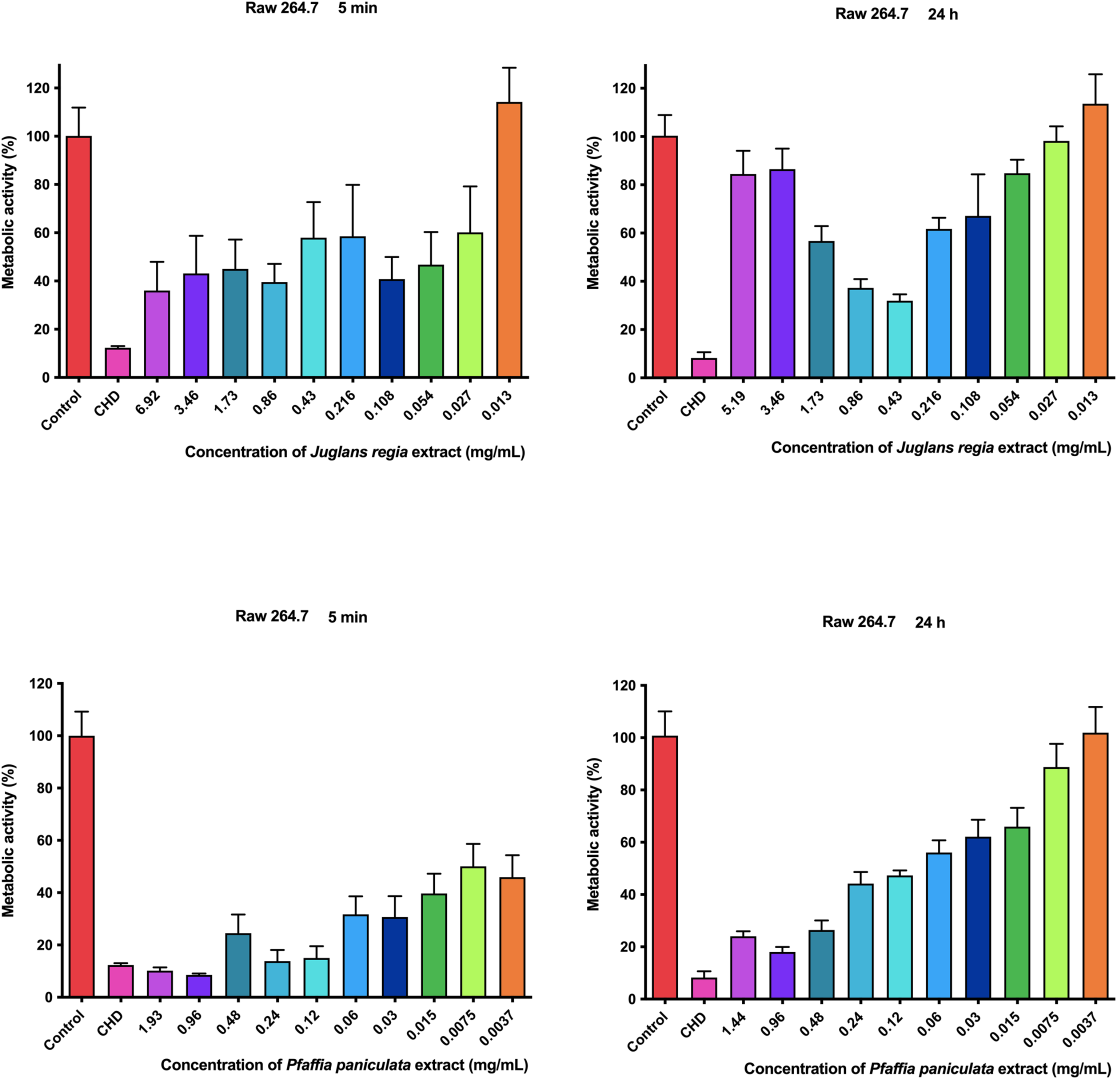
Antibiofilm action of *J. regia* and *P. paniculata* extracts at 5 min and 24h.

The results of the antibiofilm activity of *J. regia* and *P. paniculata* extracts on *P. endodontalis* and *P. gingivalis* monotypic biofilms are presented in **Figure 3**. *J. regia* extract reduced *P. endodontalis* and *P. gingivalis* biofilms by over 96% for all concentrations and durations tested. *P. paniculata* extract showed a reduction of more than 99% for all groups, except for the concentration of 0.86 mg/mL at 24h which showed a reduction of 89%. No statistical difference was observed between the concentrations tested.

### 3.4 Metabolic activity assessment of *J. regia* and *P. paniculata* extracts on RAW 264.7 mouse macrophage

**Figure 4:**
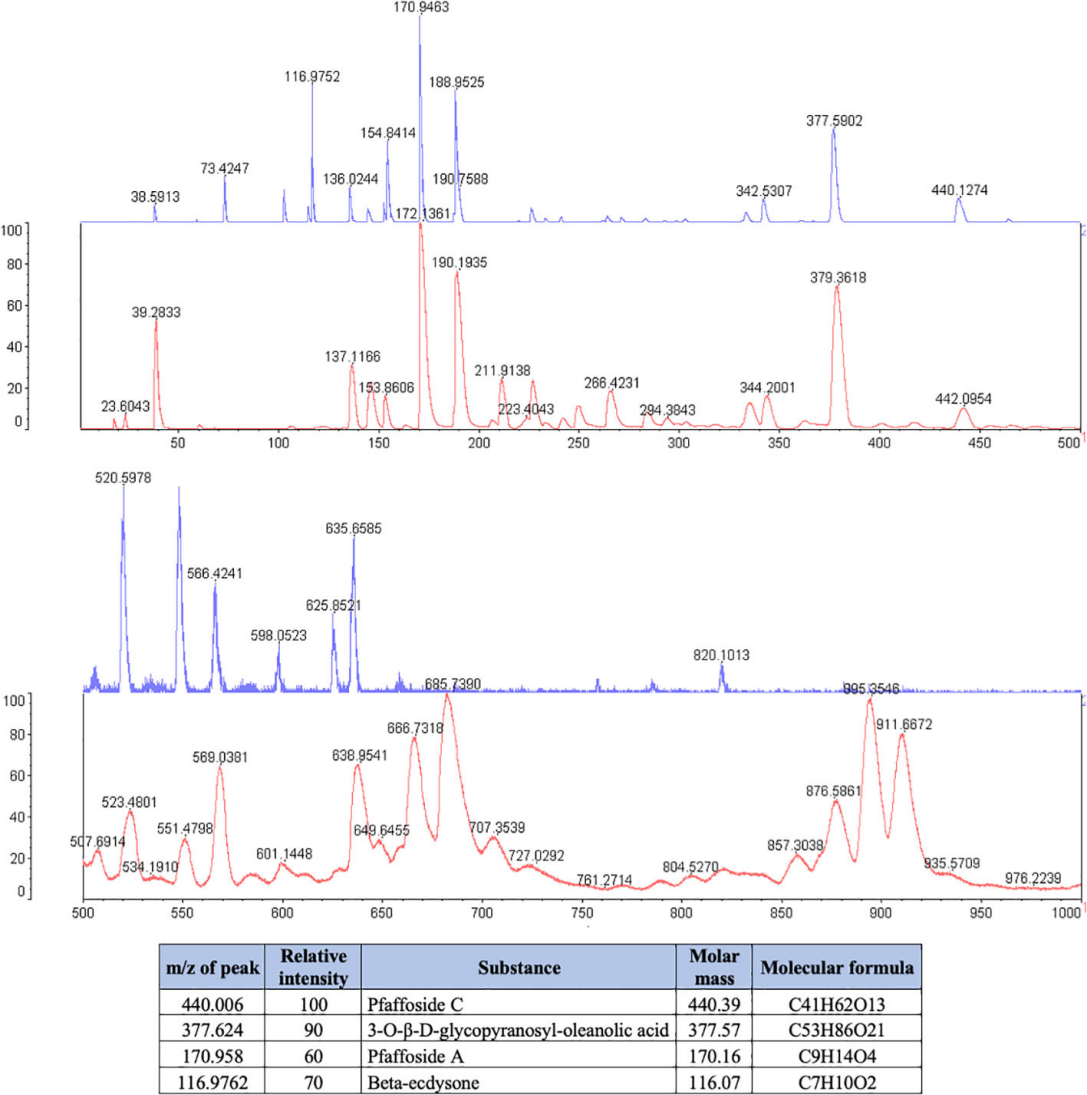
Metabolic activity of *J. regia* and *P. paniculata* on Raw 264.7.

The results of the metabolic activity of *J. regia* and *P. paniculata* extracts on RAW 264.7 mouse macrophage are presented in **Figure 4**

### 3.5 Genotoxity of *J. regia* and *P. paniculata* on mouse macrophages (RAW 264.7)

**Figure 5:**
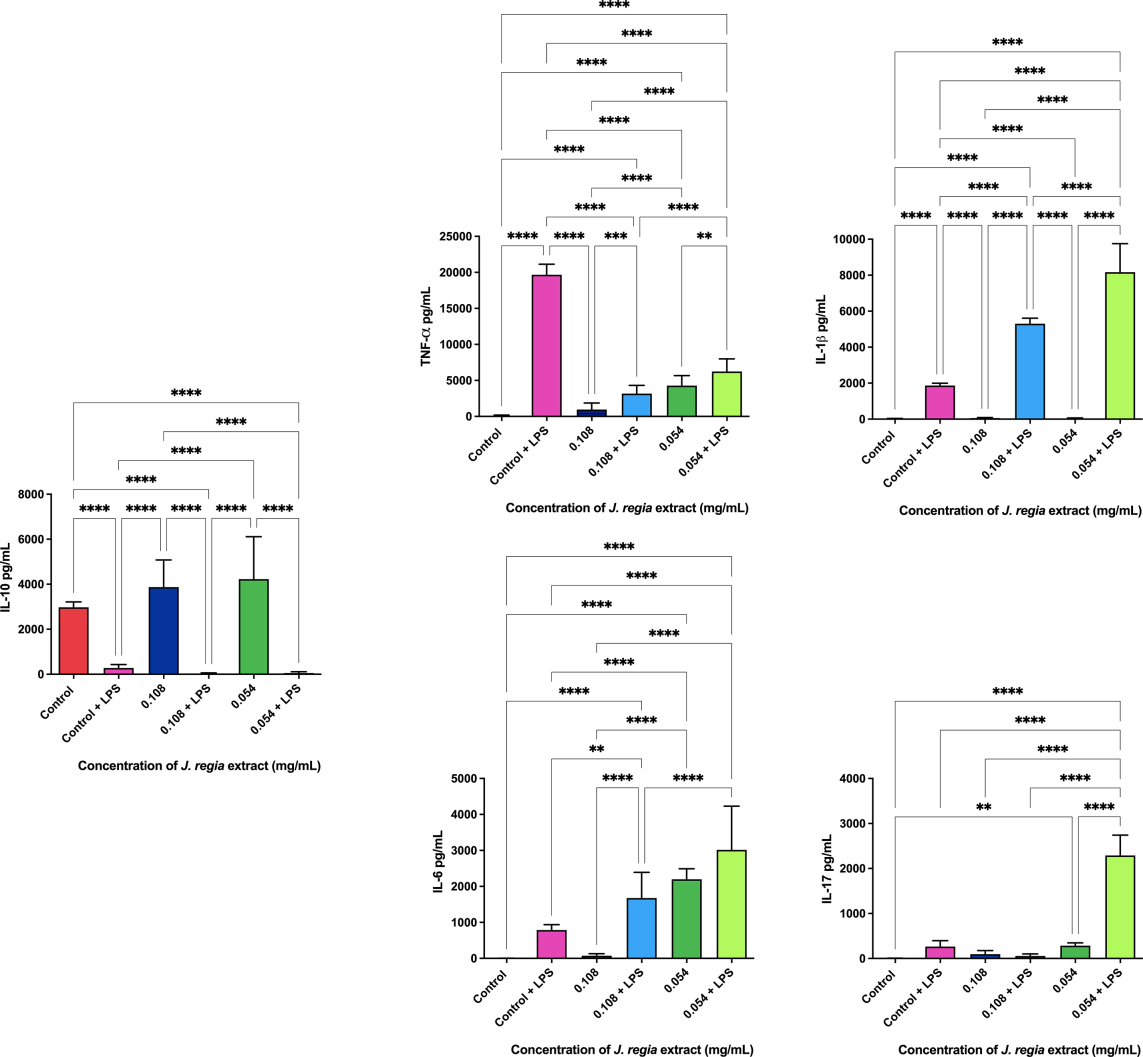
Genotoxity assay of *J. regia* and *P. paniculata* extracts on RAW 264.7 cells. p < 0.0021 (**), p < 0.0002 (***), p < 0.0001 (****).

The results of the micronucleus test of *J. regia* and *P. paniculata* on mouse macrophages (RAW 264.7) are presented in **Figure 5**.

Application of *J. regia* extract at concentrations of 0.108 and 0.054 mg/mL for 24h on mouse macrophage (RAW 264.7) cells resulted in the formation of 13 and 7 micronuclei, respectively, in a total count of 2,000 cells. Concentrations of 0.48 mg/mL and 0.24 mg/mL showed statistically similar effects to those of the control group (DMEM), but significantly different from those of the EMS group.

Application of *P. paniculata* extract at concentrations of 0.48 and 0.24 mg/mL for 24h on mouse macrophage (RAW 264.7) cells resulted in the formation of 8 and 10 micronuclei, respectively, in a total count of 2,000 cells. Concentrations of 0.48 mg/mL and 0.24 mg/mL showed statistically similar effects to those of the control group (DMEM), but significantly different from those of the EMS group.

Legend: The micrographs are represented by the heatmap rows, while the columns represent the groups tested. The number of micronuclei is shown in color, with blue (0) being the best score and yellow (7) the worst.

### 3.6 Immunological analysis of *J. regia* and *P. paniculata* extracts on mouse macrophage (RAW 264.7)

**Figure 6:**
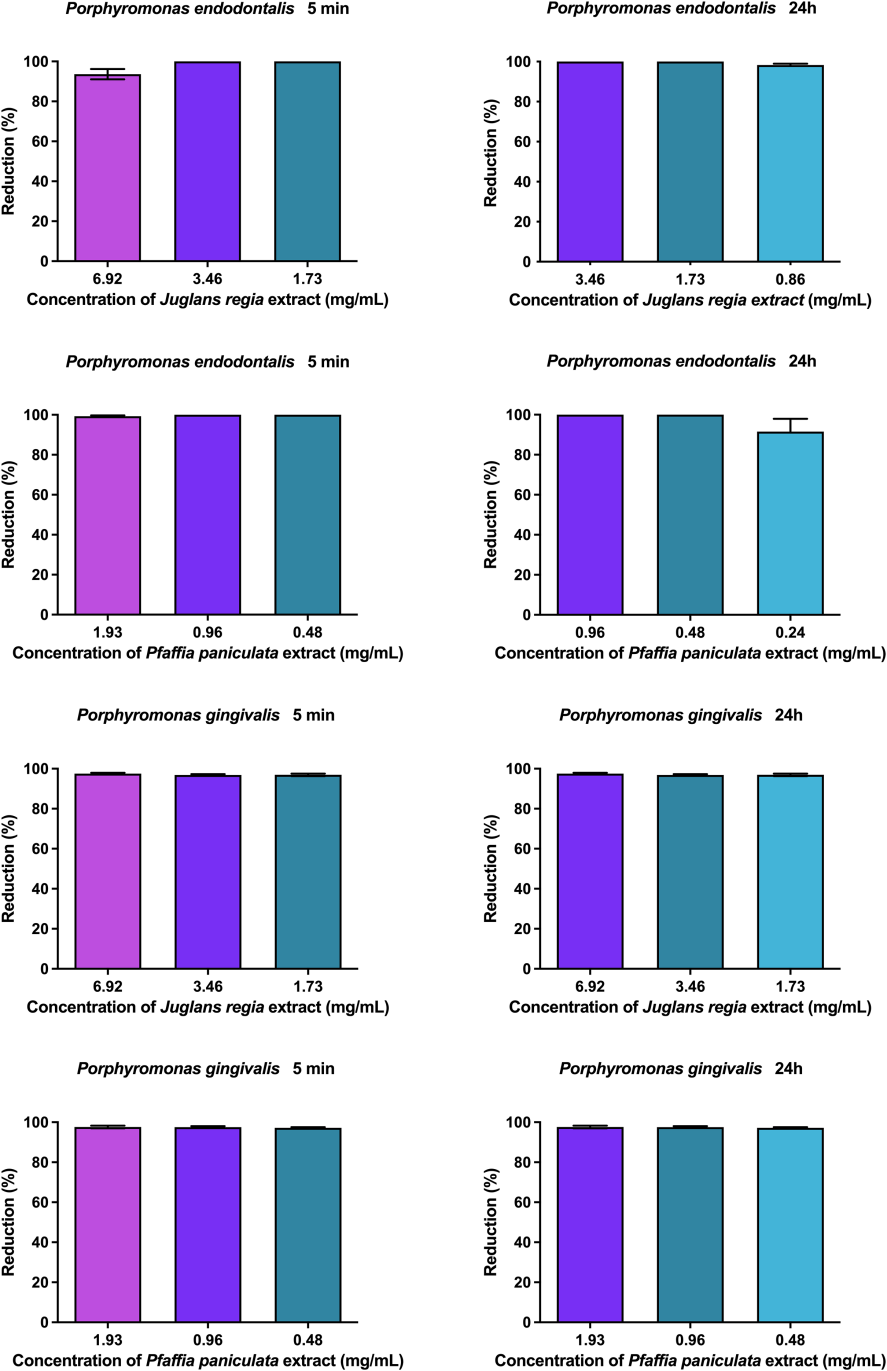
*J. regia* ELISA immunoassay.

Legend: p < 0.0021 (**), p < 0.0002 (***), p < 0.0001 (****).

The ELISA immunoenzymatic test revealed that the administration of 0.108 and 0.054 mg/ml concentrations of *J. regia* extract promoted the production of 3873.3 and 4226.6 pg/ml of the anti-inflammatory cytokine IL-10. The dosage of pro-inflammatory cytokines with and without the application of the extracts is shown in **Figure 6**

**Figure 7:**
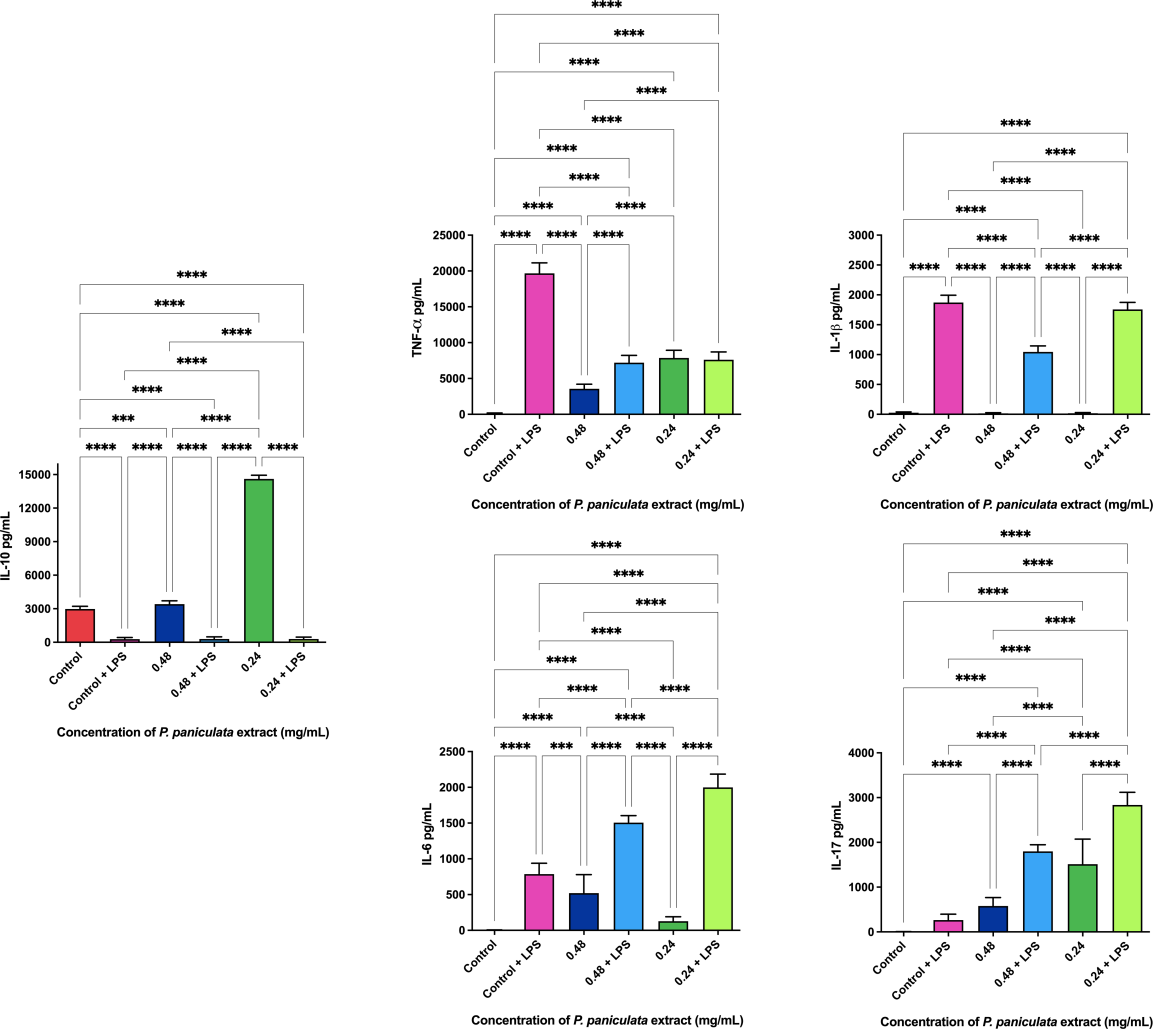
*P. paniculata* ELISA immunoassay. p < 0.0002 (***), p < 0.0001 (****).

Legend: p < 0.0021 (**), p < 0.0002 (***), p < 0.0001 (****).

The ELISA immunoenzymatic test revealed that the administration of 0.48 and 0.24 mg/ml concentrations of *P. paniculata* extract promoted the production of 3416.6 and 14606.6 pg/ml of the anti-inflammatory cytokine IL-10. The dosage of pro-inflammatory cytokines with and without the application of the extracts is shown in **Figure 7**

## 4 Discussion

Periodontal disease (PD) remains a major global public health concern, affecting 50% of the worldwide population and imposing substantial clinical and economic burdens (Jin et al., 2016; Word Health Organization, 2022). Emerging evidence highlights a pathogenic link between PD and Alzheimer’s disease (AD), with Porphyromonas gingivalis identified as a key mediator in neuroinflammatory pathways that drive neurodegeneration (Ilievski et al., 2018; Li et al., 2024; Qiu et al., 2024). Specifically, *P. gingivalis* has been shown to induce amyloid-β (Aβ) plaque deposition, a pathological hallmark of AD, via chronic inflammatory mechanisms (Ilievski et al., 2018). These insights underscore the urgent need for innovative PD therapies that may also mitigate AD-associated risks.

In our study, *J. regia* extracts and *P. paniculata* extracts demonstrated important antimicrobial action against the anaerobic bacteria *P. endodontalis and P. gingivalis*, two key anaerobic bacteria implicated in PD. Our results demonstrated that both extracts effectively inhibited planktonic cultures of these bacteria and reduced biofilm formation by more than 90%. These findings are of significant value, given the notorious difficulty in treating biofilms, which are highly resistant to conventional antimicrobial therapies. Mohammed et al. previously reported the antimicrobial activity of *J. regia* against *P. gingivalis*, further corroborating our results by showing inhibition of bacterial growth through interference with key metabolic pathways (Mohammed et al., 2023). Although antimicrobial studies on *P. paniculata* are relatively scarce, our research group has contributed to this field. In previous studies, Miranda DG et al (Miranda et al., 2024a; Miranda et al., 2024b). demonstrated the activity of the glycolic extract of *P. paniculata* against several aerobic bacteria, including *Staphylococcus aureus, Staphylococcus epidermidis, Pseudomonas aeruginosa* and *Streptococcus mutans*, as well as its antifungal efficacy against *Candida* species. These results emphasize the broad-spectrum antibacterial potential of *P. paniculata*, which is attributed to its phytochemical constituents, particularly pfaffosides A and C, 3-O-β-D-glycopyranosyl-oleanolic acid, and Beta-ecdysone.

The phytochemical composition of *J. regia* extract contributes to their potent antimicrobial activity. *J. regia* contains compounds such as phytoconstituents Regiolone, Miquelianin and Gallic Acid which are known for their antimicrobial properties against a range of pathogenic bacteria. Firstly, Regiolone has shown significant antimicrobial activity, as demonstrated by Zakavi et al., against gram-positive oral pathogens such as *Streptococcus mutans, Streptococcus salivarius, Streptococcus sanguis*, and *Staphylococcus aureus* (Zakavi et al., 2013), highlighting its broad-spectrum potential. Furthermore, Regiolone has also exhibited antifungal properties, further supporting its versatility as a potential therapeutic agent (Xu et al., 2021). Secondly, Miquelianin has demonstrated potential antimicrobial activity, though its exact mechanism remains to be fully elucidated. In studies with rats on a high-calorie diet, Miquelianin was shown to modulate the gut microbiota, promoting the growth of beneficial bacteria such as *Bacteroides, Akkermansia*, and *Mucispirillum*, while reducing the prevalence of harmful bacteria like *Lachnospiraceae, Faecalibaculum, Roseburia*, and *Colidextribacter* (Wang et al., 2023). This microbial modulation suggests that Miquelianin plays a role in maintaining microbial balance, likely through its antimicrobial properties. Thirdly, the presence of Gallic Acid, another key component with well-documented antimicrobial effects against various pathogens (Tian et al., 2022; Zhang et al., 2022), further supports these findings.

Furthermore, the anti-inflammatory properties of both *J. regia* and *P. paniculata* extracts were explored in this study. Inflammation plays a central role in both periodontal disease (PD) and Alzheimer’s disease (AD), with chronic oral infections contributing to systemic inflammation and worsening neurodegenerative processes. Our findings showed that both extracts significantly reduced pro-inflammatory cytokines, including TNF-α, IL-1β, and IL-6, in LPS-stimulated macrophages, while promoting the production of the anti-inflammatory cytokine IL-10.

These results are in line with previous studies. Wang et al (Wang et al., 2020). demonstrated that *J. regia* extract reduced TNF-α, IL-1β, and IL-6 levels in LPS-stimulated rats, while also decreasing oxidative stress through reduced mRNA synthesis of pro-inflammatory cytokines. Our study confirms the reduction of TNF-α, though we observed a less pronounced effect on IL-1β and IL-6, indicating the need for further research to clarify *J. regia*’s full anti-inflammatory potential.

The anti-inflammatory action of *P. paniculata* was similarly supported by Costa et al (Costa et al., 2015), who observed a decrease in TNF-α, IL-1β, and IL-6 in a rat model of intestinal inflammation, with results comparable to prednisolone treatment. In our study, *P. paniculata* reduced IL-1β and increased IL-10 production, but showed an unexpected increase in TNF-α levels, which contrasts with Costa et al.’s findings. This suggests that *P. paniculata*’s effects may vary depending on the inflammation model used, warranting further investigation into its precise immunomodulatory mechanisms.

Knowing this, the study by Ilievski et al (Ilievski et al., 2018). provides additional insight into how chronic infections with *P. gingivalis* can exacerbate neuroinflammation and contribute to neurodegenerative processes. In their work, rats infected with *P. gingivalis* for 22 weeks exhibited increased expression of gingipain enzymes within hippocampal cells, alongside elevated levels of pro-inflammatory cytokines such as TNF-α, IL-1β, and IL-6. This inflammatory response was associated with the production of β42 amyloid plaques, a hallmark of Alzheimer’s disease, further linking periodontal infections to neurodegeneration (Monsalvo-Maraver et al., 2023; Zhang et al., 2023). The current study’s findings are particularly relevant, as *J. regia* and *P. paniculata* extracts demonstrated significant anti-inflammatory effects, reducing TNF-α levels and inhibiting *P. gingivalis* biofilms (Jin et al., 2016; Arastu-Kapur et al., 2020; Word Health Organization, 2022), which could play a role in mitigating both periodontal disease and the associated risk of Alzheimer’s.

Given the confirmed biological activities of *J. regia and P. paniculata*, assessing their cellular toxicity is essential for their potential development as therapeutic agents. In the present study, *P. paniculata* extract demonstrated toxicity in RAW 264–7 macrophage cells, with cell viability below 50% after 5 min of exposure. However, after 24 hours, cell viability remained above 50% at concentrations lower than 12.5 mg/mL. When comparing these findings with the literature, only two relevant studies were found regarding the cytotoxicity of *P. paniculata*. In the study by Eberlin et al (Eberlin et al., 2009), a dermatological cream containing *P. paniculata* extract along with *Ptychopetalum olacoides* and *Lilium candidum* was tested on 21 healthy individuals aged between 20 and 55 years. The cream, designed to reduce dark spots around the eyes, demonstrated significant anti-inflammatory effects without showing any toxicity in the participants. This supports the notion that *P. paniculata* can be safe when applied topically, though further studies are needed to fully assess its safety for other applications (Mozar et al., 2015).

## 5 Conclusion

This study demonstrates that *J. regia* and *P. paniculata* extracts exhibit dual therapeutic potential combating periodontal pathogens while potentially mitigating neurodegenerative risks linked to chronic inflammation. The antimicrobial Efficacy was showed >90% biofilm reduction against *Porphyromonas endodontalis* and *P. gingivalis*, Minimum inhibitory concentrations (MICs) and bactericidal effects (MBCs). Anti-Inflammatory Activity with Downregulation of pro-inflammatory cytokines (TNF-α, IL-1β, IL-6) and Upregulation of anti-inflammatory IL-10. Safety Profile for *J. regia* extract and Dose-dependent cytotoxicity at higher concentrations by *P. paniculata* extract. However, *P. paniculata* remains a promising candidate due to its established safety in topical applications, suggesting potential for further therapeutic development with appropriate dosage considerations. These findings underscore the potential of these extracts in addressing for Oral microbiome modulation (targeting dysbiosis) and Systemic inflammation control (potentially reducing AD risk), warranting further research into Elucidate molecular mechanisms, Optimize dosage formulations to balance efficacy and safety and Validate effects in *in vivo* models and clinical trials.

## Data Availability

The raw data supporting the conclusions of this article will be made available by the authors, without undue reservation.
