# Correction: *Juglans regia* and *Pfaffia paniculata* extracts: implications for periodontal disease treatment and correlation with Alzheimer’s risk

**DOI:** 10.3389/fcimb.2026.1850828

**Published:** 2026-05-08

**Authors:** Diego Garcia Miranda, Florence Carrouel, Nina Attik, Gabriela Ferraz Araujo, Nicole Fernanda Dos Santos Lopes, Maria Cristina Marcucci, Flavia Pires Rodrigues, Giovanna Arruda Caires, Hugo Vigerelli, Bruno Henrique Godoi, Cristina Pacheco-Soares, Lucas de Paula Ramos

**Affiliations:** 1 Laboratory Health Systemic Process - P2S, UR4129, Faculty of Medicine Laennec, University Claude Bernard Lyon 1, University of Lyon, Lyon, France; 2 Department of Biosciences and Oral Diagnosis, Institute of Science and Technology, São Paulo State University, São José dos Campos, Brazil; 3 Multimaterials and Interfaces Laboratory, CNRS UMR 5615, University Claude Bernard Lyon 1, University of Lyon, Lyon, France; 4 Faculty of Medicine and Health, School of Dentistry, Oral Biology Division, University of Leeds, Leeds, United Kingdom; 5 Laboratory of Genetics, Butantan Institute, São Paulo, Brazil; 6 Laboratório de Bioquímica, Instituto Butantan, São Paulo, Brazil; 7 Laboratory of Cell Compartment Dynamics, Institute of Research and Development, University of Vale do Paraíba, São José dos Campos, Brazil; 8 School of Dentistry, Federal University of Alfenas—UNIFAL, Alfenas, Brazil

**Keywords:** *Porphyromonas endodontalis*, neurodegenerative disease, dementia, herbal medicine, gram-negative anaerobes, inflammation, antimicrobial agents

There was a mistake in **Figure 1** as published. **Figure 1** should show the phytochemical analysis of the compounds, but it displays the graph from **Figure 5** (genotoxicity analysis). The correct figure that should appear as **Figure 1** is shown in **Figure 2**.

**Figure 1:**
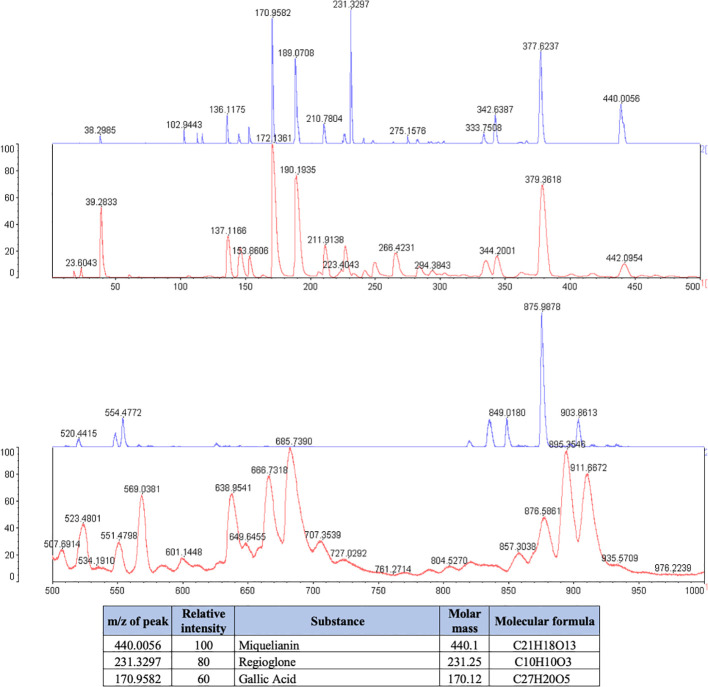
Phytochemical spectrum of the *Juglans regia* glycolic extract.

**Figure 2:**
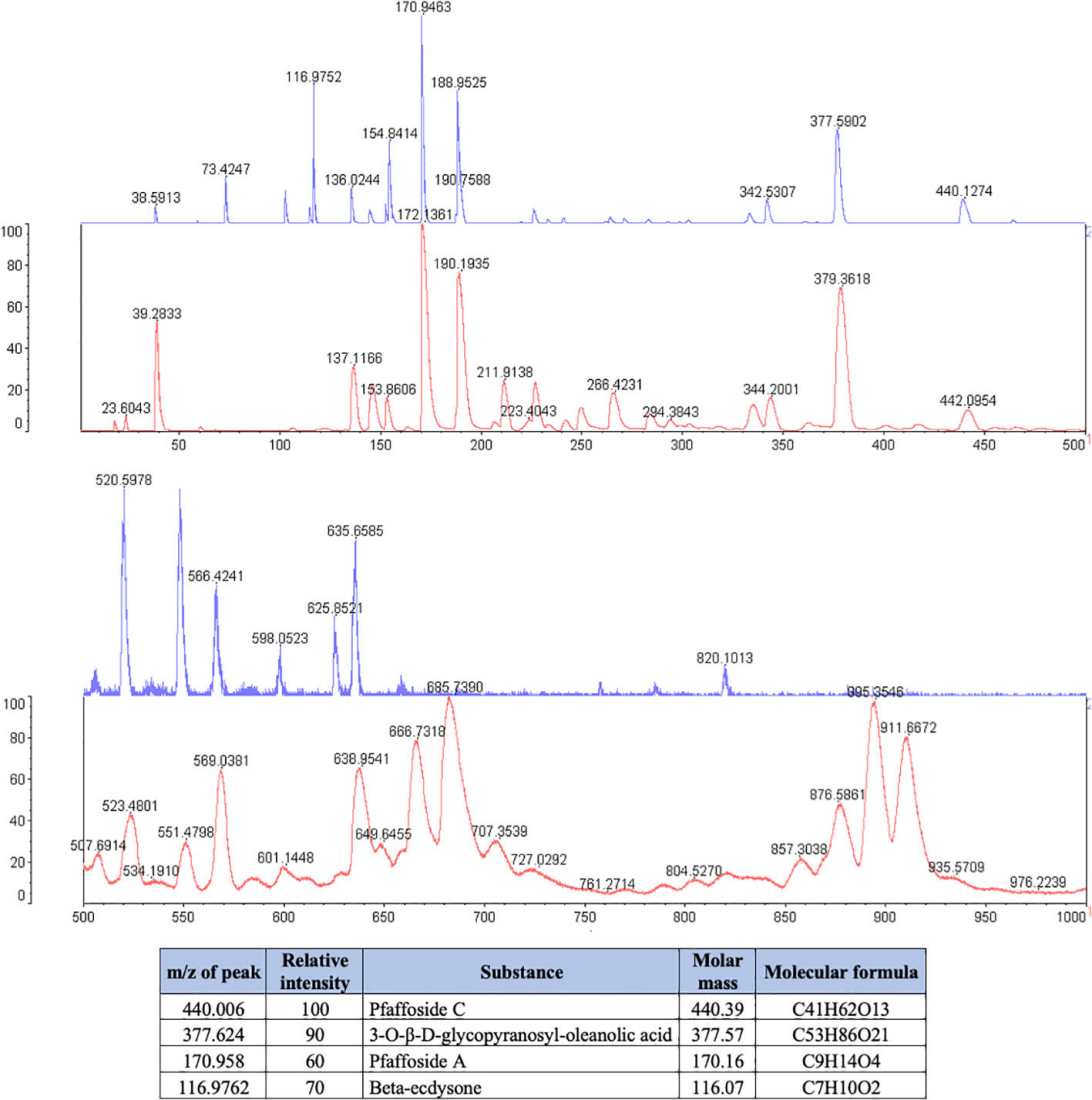
Phytochemical spectrum of the *Pfaffia paniculata* glycolic extract.

There was a mistake in **Figure 2** as published. **Figure 2** should show the Antibiofilm action of J. regia and P. paniculata. The correct figure that should appear as **Figure 2** is shown in **Figure 4**.

There was a mistake in **Figure 3** as published. **Figure 3** should show the phytochemical analysis of the compounds by P. paniculata. The correct figure that should appear as **Figure 3** is shown in **Figure 6**.

**Figure 3:**
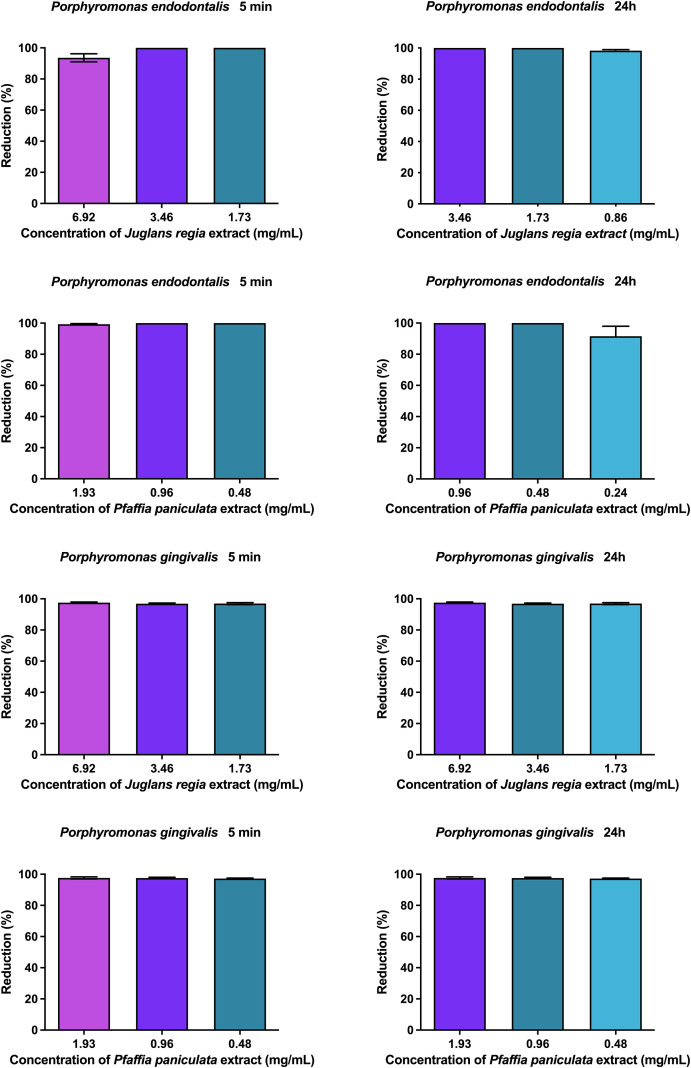
Antibiofilm action of *J. regia* and *P. paniculata* extracts at 5 min and 24h.

There was a mistake in **Figure 4** as published. **Figure 4** should show the Metabolic activity of J. regia and P. paniculata on Raw 264.7. The correct figure that should appear as **Figure 4** is shown in **Figure 3**.

**Figure 4:**
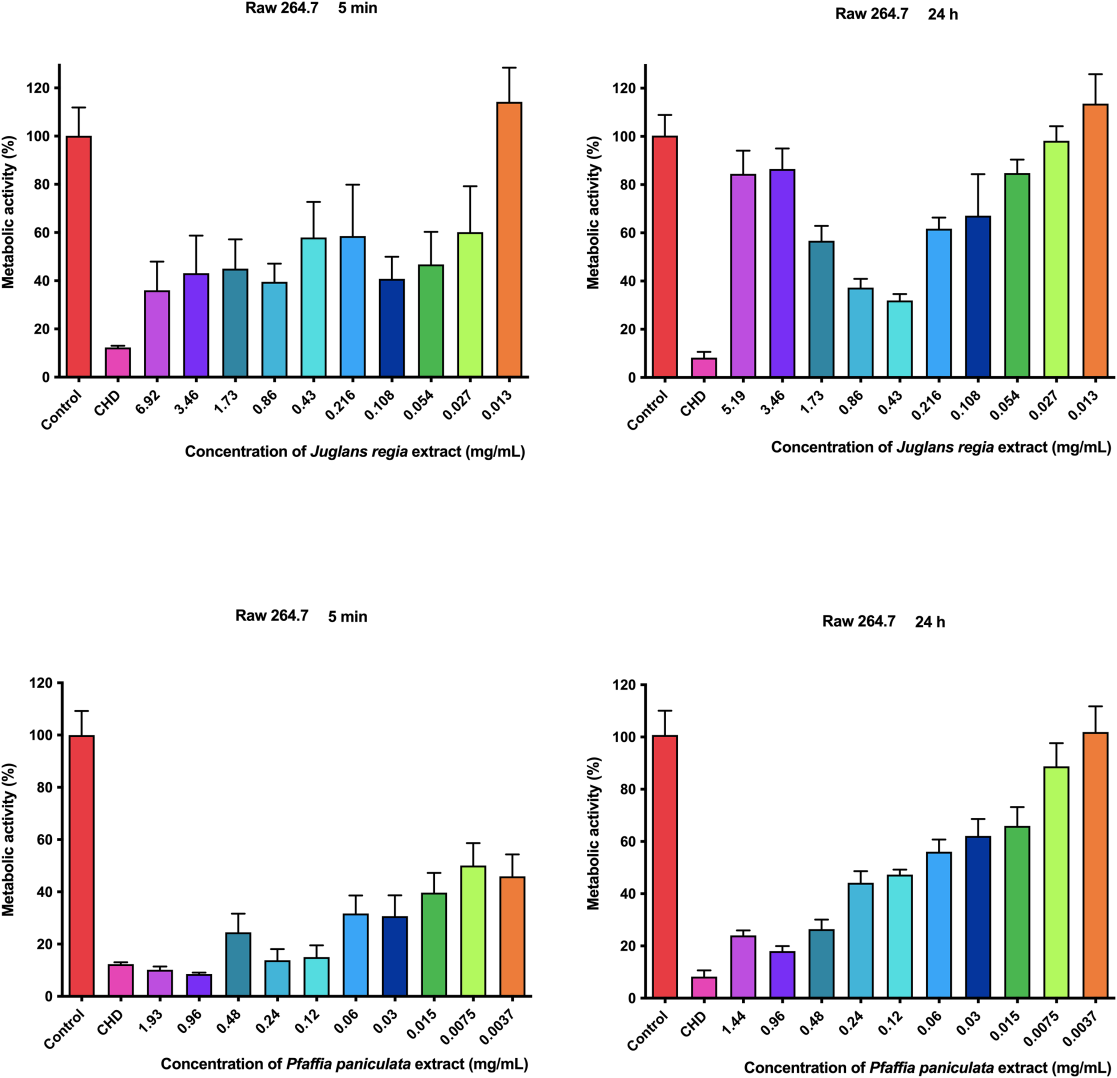
Metabolic activity of *J. regia* and *P. paniculata* on Raw 264.7.

There was a mistake in **Figure 5** as published. **Figure 5** should show the Genotoxity assay. The correct figure that should appear as **Figure 5** is shown in **Figure 1**.

**Figure 5:**
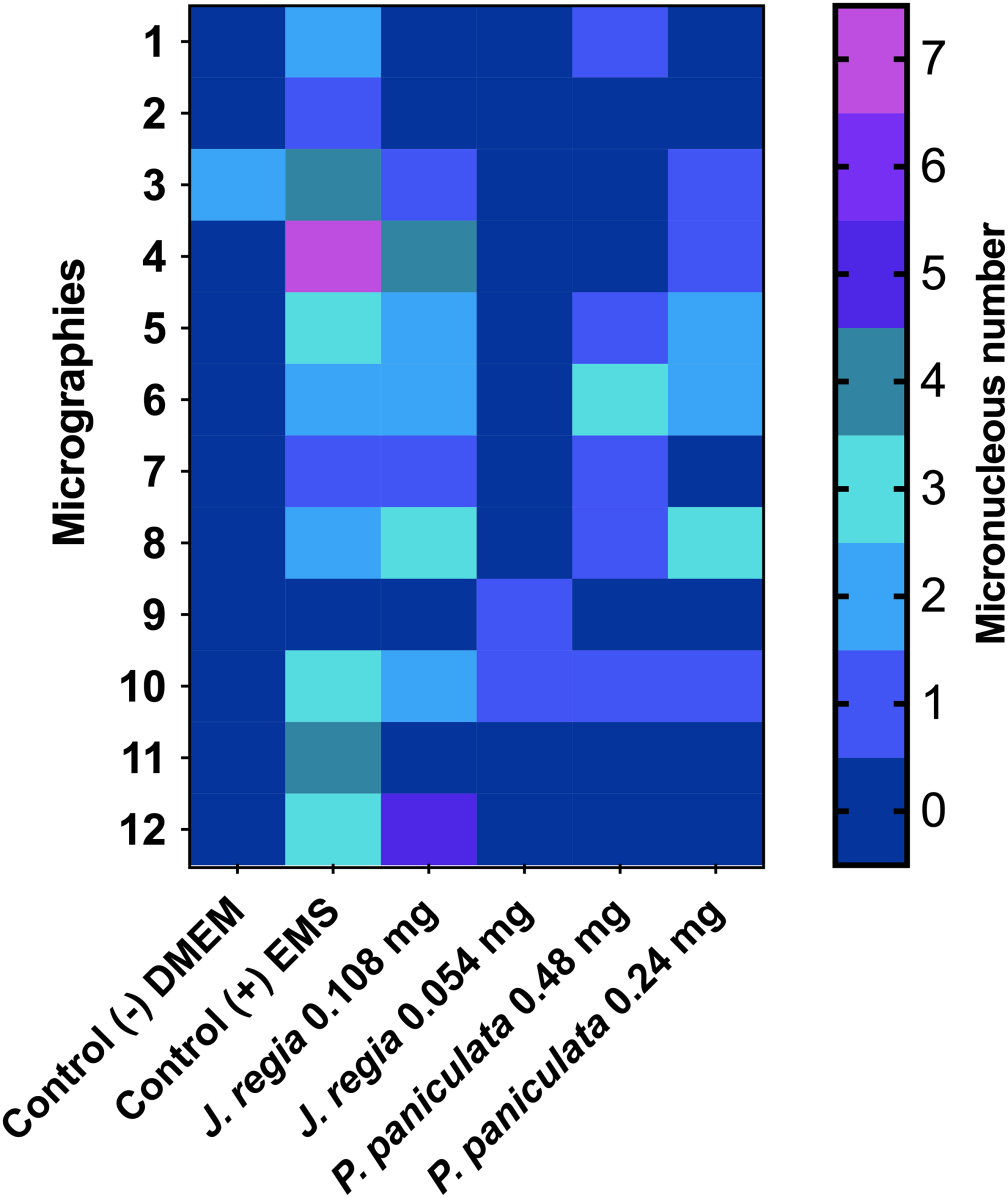
Genotoxity assay of *J. regia* and *P. paniculata* extracts on RAW 264.7 cells. p < 0.0021 (**), p < 0.0002 (***), p < 0.0001 (****).

There was a mistake in **Figure 6** as published. **Figure 6** should show the J. regia ELISA immunoassay. The correct figure that should appear as **Figure 6** is shown in **Figure 5**.

**Figure 6:**
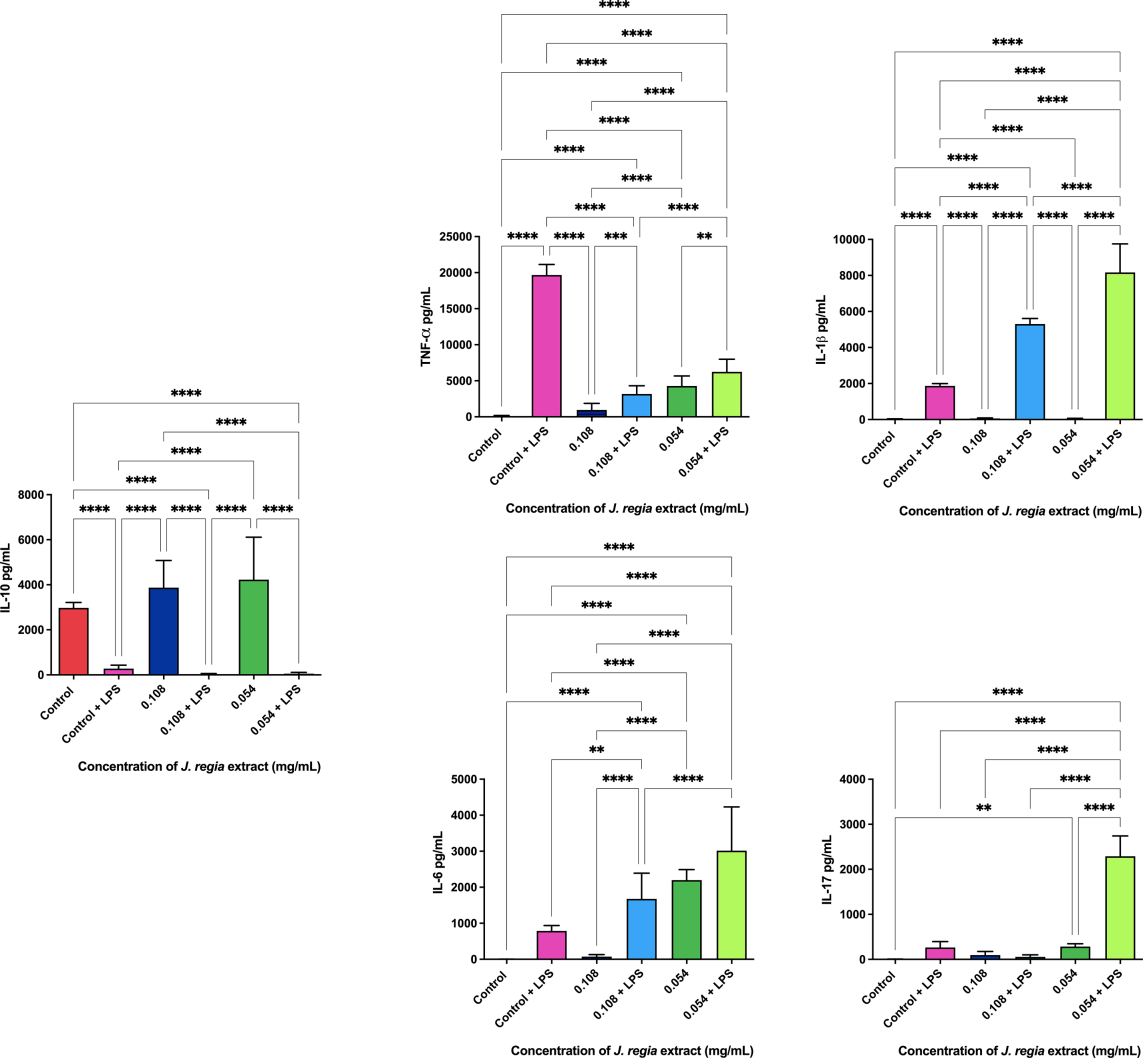
*J. regia* ELISA immunoassay.

